# Insects Can Count: Sensory Basis of Host Discrimination in Parasitoid Wasps Revealed

**DOI:** 10.1371/journal.pone.0138045

**Published:** 2015-10-14

**Authors:** Sara Ruschioni, Joop J. A. van Loon, Hans M. Smid, Joop C. van Lenteren

**Affiliations:** 1 Laboratory of Entomology, Wageningen University, Wageningen, The Netherlands; 2 Dipartimento di Scienze Agrarie, Alimentari ed Ambientali, Università Politecnica delle Marche, Ancona, Italy; 3 Laboratory for Chemical Ecology and Insect Behavior, Department of Entomology and Acarology, ESALQ, University of Sao Paolo, Piracicaba, SP, Brazil; AgroParisTech, FRANCE

## Abstract

The solitary parasitoid *Leptopilina heterotoma* is one of the best studied organisms concerning the ecology, behaviour and physiology of host discrimination. Behavioural evidence shows that *L*. *heterotoma* uses its ovipositor to discriminate not only between parasitized and unparasitized *Drosophila melanogaster* larvae, but also to discriminate between hosts with different numbers of parasitoid eggs. The existing knowledge about how and when the parasitoid marks the host motivated us to unravel the chemosensory basis of host discrimination by *L*. *heterotoma* that allows it to choose the “best” host available. In this paper we report on electrophysiological recordings of multi-neural responses from the single taste sensillum on the tip of the unpaired ovipositor valve. We stimulated this sensillum with haemolymph of unparasitized, one-time-parasitized and two-times-parasitized *Drosophila* larvae. We demonstrate for the first time that quantitative characteristics of the neural responses to these haemolymph samples differed significantly, implying that host discrimination is encoded by taste receptor neurons in the multi-neuron coeloconic ovipositor sensillum. The activity of three of the six neurons present in the sensillum suffices for host discrimination and support the hypothesis that *L*. *heterotoma* females employ an ensemble code of parasitization status of the host.

## Introduction

Insect parasitoids lay their eggs in, on or near the body of their hosts. Parasitoid larvae are entirely dependent on the host for their development, as they feed exclusively from its tissues until they emerge as adults. Fitness of parasitoids is therefore strongly dependent on the quality of the host and, thus, on the host selection decisions made by the ovipositing female [1]. In solitary parasitoids only a single larva can develop inside the host. If a solitary parasitoid female oviposits in an already parasitized host, a behaviour called superparasitism [2], the ensuing larval competition ends in the death of the supernumerary larvae [1, 3–7]. For this reason superparasitism should be strongly selected against, since it costs eggs (offspring) and/or time [8]. One of the mechanisms to prevent superparasitism is that the parasitoid discriminates between parasitized and unparasitized host larvae and lays only eggs in unparasitized hosts. Host discrimination is known to occur in many parasitoid species [9–15], non-discriminating parasitoid species are rare [4, 8, 9].

One of the best studied organisms concerning the ecology, behaviour and physiology of host discrimination is the solitary parasitoid *Leptopilina heterotoma* (Thomson, 1862) (Hymenoptera: Figitidae) which uses larvae of *Drosophila* spp. (Diptera: Drosophilidae) as hosts [16–18] including the recent invasive species *Drosophila suzukii* [19–20]. Interestingly, *L*. *heterotoma* is not only able to discriminate between unparasitized and parasitized hosts, but also between hosts with different numbers of parasitoid eggs. This phenomenon of ‘counting’ is known for a few other solitary [21] and gregarious [22–26] parasitoids.

Discrimination behaviour was hypothesized to be based on information mediated by sensilla on the parasitoid ovipositor [9, 27]. Earlier attempts using sensory physiological methods to study the basis of host discrimination failed [9, 28, 29]. Nevertheless, behavioural observations suggested that only gustatory receptors on the parasitoid ovipositor are used for host discrimination by *L*. *heterotoma* [9]. In fact, the decision on acceptance or rejection of a host by *L*. *heterotoma* occurs in the first seconds after inserting the ovipositor into the host [9, 30].

The ovipositor of *L*. *heterotoma* carries seven gustatory sensilla at the distal end, three on each of the paired valves and one on the unpaired valve. These gustatory sensilla are all innervated by six neurons [31]. The sensillum on the unpaired valve is asymmetrically placed at the very tip of the unpaired valve just where the rachis ends [31]. We previously reported on multi-neural responses from the single sensillum on the unpaired ovipositor valve, but upon stimulation with unparasitized and parasitized host haemolymph we found no significant differences in summed response frequencies [31]. The behavioural evidence that the ovipositor is used to discriminate between parasitized and unparasitized hosts together with the information we have about how and when the host is marked by the parasitoid [2, 30, 32] motivated us to re-assess the chemosensory basis of host discrimination by *L*. *heterotoma* that allows it to choose the “best” host available.

In this paper we report about recordings of multi-neural responses from the single sensillum on the unpaired ovipositor valve. This time, we stimulated the sensillum with haemolymph of unparasitized, one-time-parasitized and two-times-parasitized *Drosophila* larvae. We demonstrate for the first time that characteristics of the neural responses to these haemolymph samples differed significantly.

## Materials and Methods

### Insects

*L*. *heterotoma* was reared in larvae of *Drosophila melanogaster* (Diptera: Drosophilidae). Rearing was done in a climate room at 25±1°C, 70% RH and L12:D12. For details on rearing of *D*. *melanogaster* see Bakker (1961, page 220), and for *L*. *heterotoma* [33], see Bakker et al. (1967, page 296) [16].

Van Lenteren (1972) observed that *L*. *heterotoma* females need experience to be able to discriminate [32]. We obtained experienced parasitoid females, by putting individual, 4–8 days old, mated *L*. *heterotoma* females into a Petri dish (diameter 45 mm; height 8 mm) with 20 *D*. *melanogaster* larvae of 2–3 days old at 25°C. When the female started to parasitize she was left with the host larvae for one hour. After this “training” period the parasitoid was considered to be “experienced”, and then kept individually in a vial at 25°C, with a small piece of wet sponge and a droplet of honey. Next day she was used to obtain one-time-parasitized host larvae (1P): *D*. *melanogaster* larvae of 3–5 days old and experienced *L*. *heterotoma* females were put together in a Petri dish with a small amount of yeast. As soon as the parasitoid laid an egg, as judged by observing the behaviour of the parasitoid [2], the parasitized larva was isolated in another Petri dish and either used for the recordings, or to prepare the two-times-parasitized host larvae (2P).

To obtain 2P, mated females of *L*. *heterotoma* were first exposed to 1P along a similar procedure as used to get experienced females. Next day, 1P larvae parasitized 10–30 minutes earlier were offered to parasitoids to obtain 2P. As soon as a parasitoid had laid a second egg in the host, the 2P were isolated and used for the recordings.

At the end of all experiments, the parasitized larvae were dissected [30] and observed at 25x magnification under strong illumination in order to determine the actual number of parasitoid eggs in the host haemolymph.

### Electrophysiology

For the stimulation procedure, the ovipositor was removed from the abdomen of a mated parasitoid by dissection at the proximal end, after which the three valves were separated. The tip-recording method for insect gustatory sensilla [34] was used. Silver wires were inserted into both glass electrodes mounted on electrode holders and connected to a DC amplifier (Taste Probe, Syntech, Hilversum, The Netherlands). The unpaired valve was partly inserted into the recording electrode (inside diameter 8.0/10.0 μm) containing Phosphate Buffered Saline (PBS) (Oxoid Limited, United Kingdom) [31]. The indifferent glass electrode (inside diameter 10.0/15.0 μm), containing PBS, was inserted into the *Drosophila* larva. Next, the coeloconic sensillum at the very tip of the unpaired valve of the *L*. *heterotoma* ovipositor was stimulated with the haemolymph of unparasitized host larvae (UP), 1P and 2P. Haemolymph oozed out of the larva as a result of inserting the indifferent glass electrode. Electrical contact was established by moving the electrode with the host larva to just touch the tip of the unpaired ovipositor valve. Contact was made within 5 sec after the larva had been mounted on the glass electrode. Once contact between the sensillum and haemolymph was achieved, it was maintained for 5 sec. The unpaired valve remains responsive for at least 5 minutes. Ten ovipositors were tested for each of the three stimuli. An ovipositor preparation was used for only one of the three stimuli.

The electrophysiological signal was imported via an IDAC interface and A/D converter (Syntech, Hilversum, The Netherlands) into a personal computer at a rate of 12.000 samples per second. Filter settings during sampling were 200–3000 Hz (-12 dB). The action potential recordings were visually analysed based on a minimal S/N ratio of 2 and biphasic shape characteristics using Autospike software release 3.7 for on-screen visualization (Syntech, Hilversum, The Netherlands).

### Data analysis

Action potential frequencies were determined by counting the spikes during the 0.2–1 sec interval after stimulation [35]. As it was not possible to assign the electrophysiological activity to individual neurons, we counted the total number of spikes. We also counted the number of doublets and triplets. Two spikes were considered a doublet if they were separated by a time interval shorter than the refractory or “silent” period [36]. Similarly, three spikes were considered a triplet when they were separated by intervals shorter than the refractory period. The numbers of spikes, doublets, triplets were counted visually. To obtain interspike interval (ISI) distribution histograms, we used fourteen 5 ms-bins. To obtain amplitude distribution histograms averaged for the 10 wasps tested each for UP, 1P and 2P recordings, we used 10 bins of 100 μV in the range between 500 and 1500 μV. ISI and amplitude distributions were constructed by using the Autospike software 3.7 measuring tools (Syntech). All of the statistical tests were performed at α = 0.05, using SPSS v. 22 (IBM, Armonk, USA).

## Results

**Fig 1:**
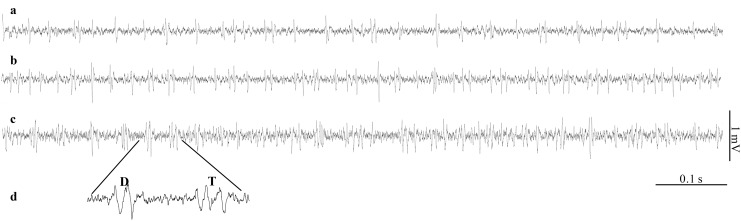
Representative recordings of electrophysiological activity of the coeloconic sensillum on the distal tip of the unpaired ovipositor valve of *Leptopilina heterotoma* to haemolymph of *Drosophila* larvae of different parasitization status, using the extracellular tip-recording technique. **(a)** Response to haemolymph of an unparasitized larva (UP). **(b)** Response to haemolymph of a larva containing one *L*. *heterotoma* egg (1P). **(c)** Response to haemolymph of a larva containing two *L*. *heterotoma* eggs (2P). **(d)** Magnified detail from c, to illustrate the occurrence of doublets (D) and triplets (T).

**Fig 2:**
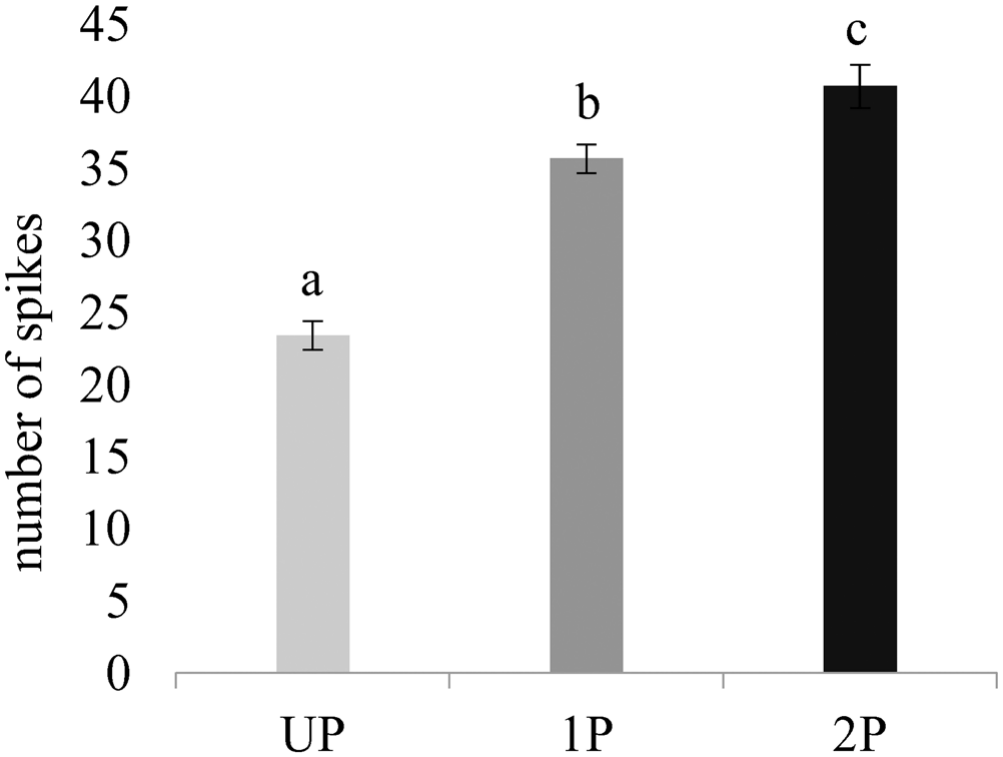
Action potential frequencies of the coeloconic sensillum on the distal tip of the unpaired ovipositor valve of *Leptopilina heterotoma*, averaged over the 10 wasps tested. Error bars are SEM (N = 10). The number of spikes was counted during the 0.2–1 sec interval after the onset of stimulation with haemolymph of UP, 1P and 2P *Drosophila*. Mean values having no letters in common differ significantly (LSD post-hoc test, *P* < 0.05).

The single sensillum on the unpaired ovipositor valve gave multi-neural responses when immersed in host haemolymph (Fig 1), but the total number of spikes differed significantly between all treatments (One-way ANOVA, F_2, 27_ = 27.25; *P* < 0.0001; Fig 2). When the sensillum was stimulated by UP haemolymph, the response frequency was significantly lower than that in response to 1P or 2P haemolymph (LSD post-hoc test, *P* < 0.05; Fig 2).

**Fig 3:**
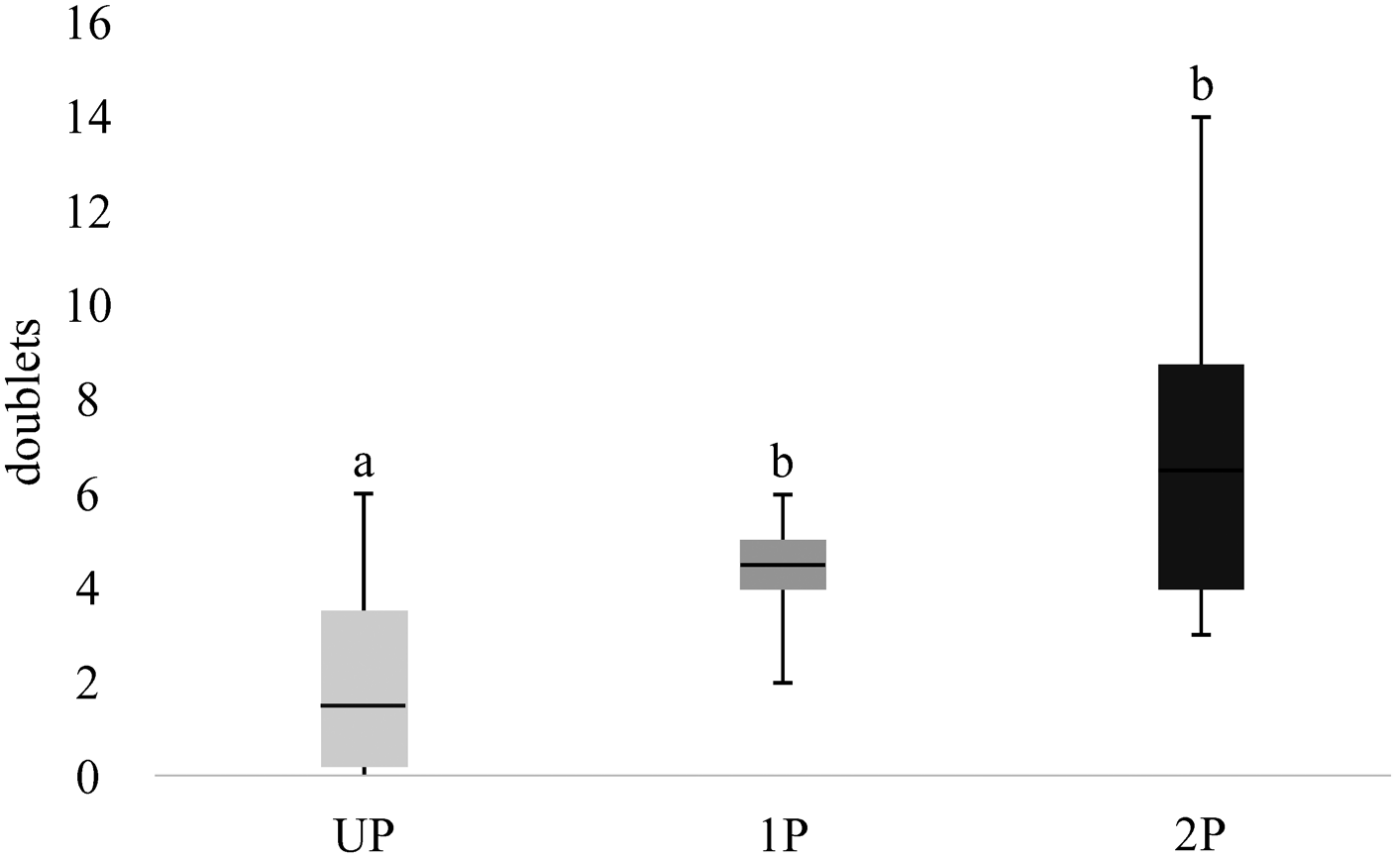
Number of doublets observed in recordings of responses to haemolymph of UP, 1P and 2P *Drosophila*, averaged over the 10 wasps tested. Error bars are SEM. The number of doublets was counted during the 0.2–1 sec interval after stimulation with haemolymphs of UP, 1P and 2P. Medians that have no letters in common differ significantly according to the Jonckheere-Terpstra test for ordered alternatives (P < 0.05).

When comparing the number of doublets found in recordings obtained upon stimulation with haemolymph of UP, 1P or 2P, significant differences were found (independent samples Jonckheere-Terpstra test, *P* = 0.001; Fig 3). No triplets were found in response to stimulation with UP haemolymph, one triplet was found in in response to 1P haemolymph, and triplets were found in 4 out of 10 recordings in response to stimulation with haemolymph of 2P.

**Fig 4:**
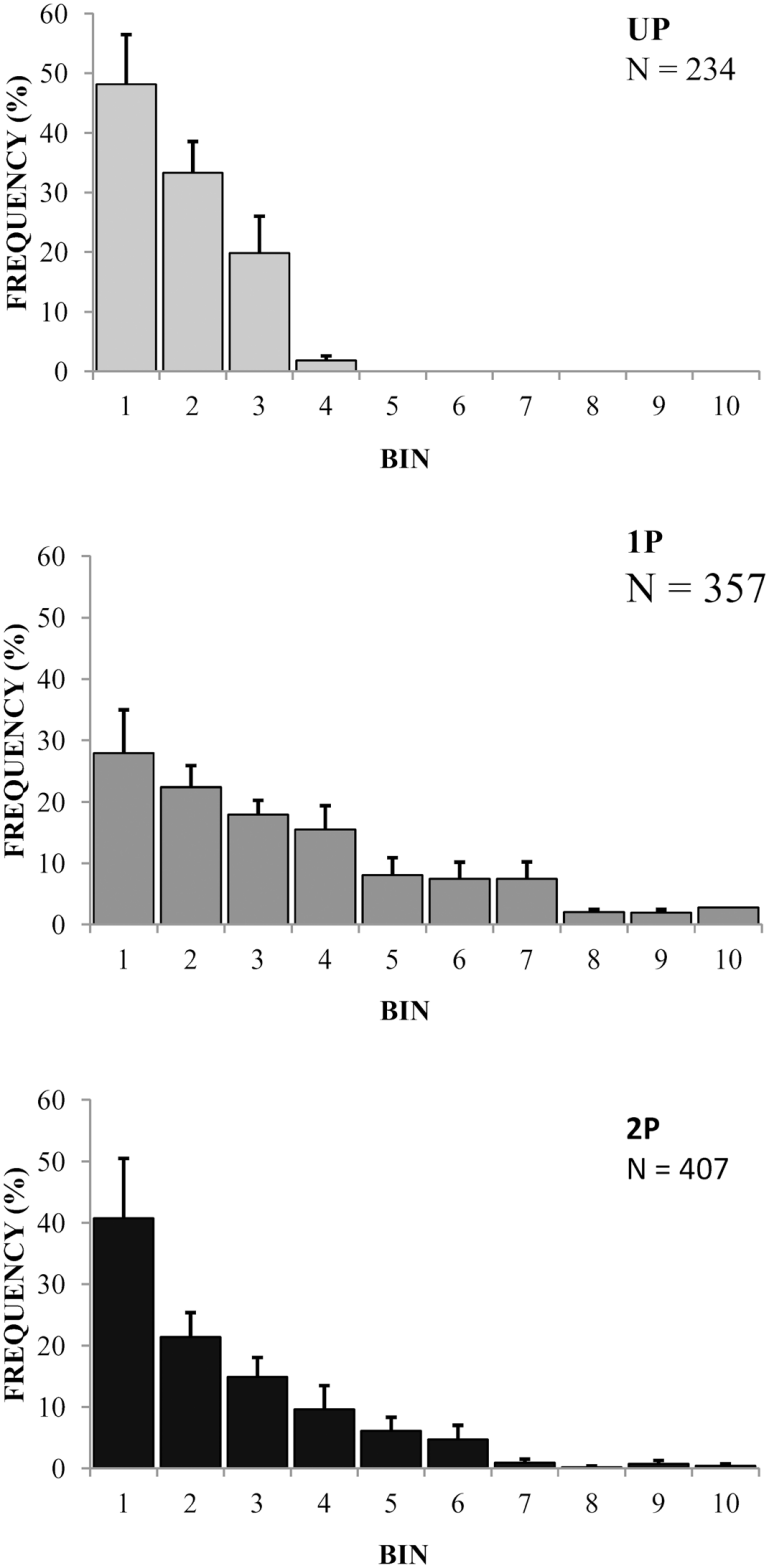
Amplitude distribution histograms for recordings in response to haemolymph from UP, 1P and 2P *D*. *melanogaster* larvae. Each spike has been assigned to a 100 μV-amplitude bin in the range between 500 and 1500 μV (bins 1–10). Mean frequency of occurrence has been plotted; error bars are SEM. N refers to the total number of spikes for recordings in response to haemolymph of UP, 1P and 2P.

Amplitude distribution histograms (Fig 4) showed spikes with amplitude from 500 mV to 1500 mV for all responses, while higher amplitudes were observed only in 1P and 2P. Frequency distributions of spike amplitudes differed significantly between recordings in response to UP, 1P and 2P haemolymph (Kruskal-Wallis test, P < 0.001; Mann-Whitney U-tests for the three pairwise comparisons; P < 0.001).

**Fig 5:**
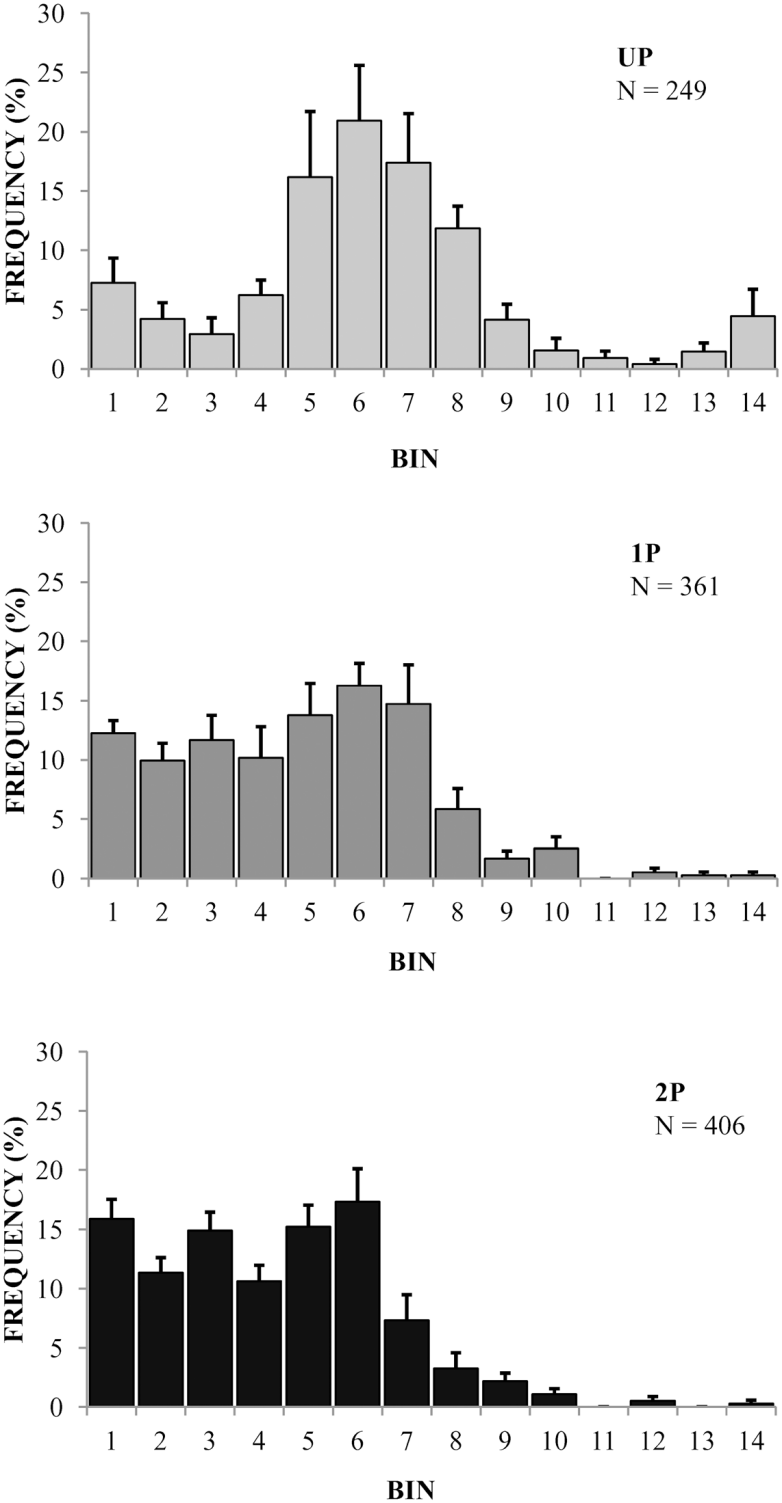
Interspike interval (ISI) distribution histogram for recordings in response to haemolymph from UP, 1P and 2P *D*. *melanogaster* larvae. Each ISI has been assigned to a 5 msec time bin in the range between 5 and 70 msec (bins 1–14). Mean frequency of occurrence has been plotted; error bars are SEM. N refers to the total number of ISIs for recordings in response to haemolymph of UP, 1P and 2P.

Frequency distributions of interspike intervals (ISIs) differed significantly between recordings in response to UP, 1P and 2P haemolymph (Kruskal-Wallis test; P = 0.029; Fig 5). The frequency distribution of ISIs in response to UP haemolymph differed significantly between UP and 1P and UP and 2P samples (Mann-Whitney U tests, P = 0.028 and P = 0.016 respectively). ISI-distributions were similar for 1P and 2P samples.

## Discussion

The discrimination behaviour of *L*. *heterotoma* is ascribed to the presence of a putative marking substance in the haemolymph released during oviposition [9]. Our electrophysiological results provide the first evidence of a chemosensory mechanism underlying the ability of *L*. *heterotoma* to detect and quantify a putative marker of the parasitized status of the host. Our data imply that the ovipositor coeloconic gustatory sensillum encodes host status with respect to parasitization, both between un-parasitized and one time- parasitized as well as between one-time and two-time parasitized hosts.

Evidence from detailed behavioural studies pointed to the crucial role of the coeloconic sensillum on the distal tip of the unpaired ovipositor valve of *L*. *heterotoma* in host discrimination. Here we identified differential neural input from this gustatory multi-neuron sensillum based on the association between distinct electrophysiological activity patterns elicited by UP, 1P and 2P haemolymph and strict behavioural discrimination.

As previously described by van Lenteren et al. (2007) the coeloconic sensillum is innervated by 6 sensory cells [31]. The multineural recordings did not allow unambiguous assignment of action potentials to individual neurons. An action potential might in principle be assigned to the neuron of origin if each neuron fires spikes with a distinct amplitude and/or shape [37]. The spike doublets and triplets recorded provide direct evidence for the simultaneous activity of at least two and three neurons, respectively [36]. More specifically, considering that we found doublets in UP, 1P and 2P, we suppose that for each stimulus at least two neurons were activated. In addition we deduce from the ISI-distributions that in case of stimulation by parasitized haemolymph, more neurons were activated than when stimulated by UP haemolymph. A higher frequency of short interspike intervals was observed for recordings in response to haemolymph of parasitized larvae, in particular of 2P. Furthermore, this hypothesis is supported by the amplitude distribution histogram (Fig 4), which shows amplitude classes above 800 mV only in response to stimulation by 1P and 2P haemolymph. We deduce from these observations that more neurons are recruited upon contact with haemolymph of parasitized larvae. An additional line of evidence is that the total number of spikes recorded differed significantly between responses to stimulation by haemolymph from UP, 1P and 2P: the higher the number of eggs in the haemolymph, the higher the frequency of firing. Taken together, these data support the hypothesis that *L*. *heterotoma* females employ an ensemble code of parasitization status of the host.

The chemical complexity of haemolymph as stimulus solution generates a multineural chemosensory activity pattern from the coeloconic sensillum, hampering unambiguous assignment of spikes to neurons. We used haemolymph because it is the natural stimulus and as such relevant for exploring gustatory coding in host discrimination [38]. The concentration of the putative marking substance indicating the presence of parasitoid eggs inside the haemocoel of the host supposedly reflects the number of eggs. Although the chemical nature of the marker is unknown, we propose that a marker substance secreted by the female at oviposition is the most likely signal for host discrimination since behavioural data have demonstrated that the signal affects host selection within seconds after oviposition [9]. Biochemical changes in the haemolymph of parasitized larvae would likely require more time to come about. The functions of host marking pheromones have been studied in several parasitoid species [12, 39], but little is known about the origin and nature of the chemical cues involved.

In addition to perceiving host marking pheromones, sensilla on the ovipositor tip have a function in the perception of kairomonal cues that influence egg-laying decisions [40–42]. These sensilla are able to evaluate the internal quality of the host [43–46], such as health of the host [47, 48], and host developmental stage [48–52], and host species [53]. The ability of a parasitoid to evaluate its hosts has vital consequences for the fitness of its offspring. In fact, with the ability to discriminate, parasitoids can prevent wastage of eggs [8, 54–57], wastage of hosts, save time and initiate migration after a number of probes in parasitized hosts [9, 55, 58].

Despite being able to discriminate it can happen that *L*. *heterotoma* superparasitizes [2]. Cases of superparasitism can be explained by several possible causes: a female lays a second egg within the period needed for building up a factor that causes avoidance of superparasitism; two or more females lay eggs simultaneously in the same hosts; a female’s tendency to oviposit increases when she encounters only parasitized hosts for a long period; or a female has not yet learned to discriminate [32]. In hymenopteran parasitoids learning seems to be extremely wide spread [59] and host discrimination has often been related to learning [60]. Van Lenteren (1972) observed that inexperienced *L*. *heterotoma* females easily lay eggs in already parasitized hosts, but stop doing this after one or a few contacts with unparasitized hosts [32].

So far, many studies described the host discrimination of parasitoids, focusing on the discrimination behaviour and on the morphology of the structures involved. With this paper, we extend the knowledge with the proof that discrimination takes places on the receptor level of a sensillum in the ovipositor. We show here that the activity of three of the six neurons present in the sensillum suffices for host discrimination. We will now start studies on discrimination among different species of hosts and/or between healthy and non-healthy (e.g. fungi or virus infected) hosts, in order to understand the functions of the other neurons in the sensillum and of the neurons in the six other chemosensilla on the tip of the ovipositor.
